# Leveraging Deep Learning to Enhance Malnutrition Detection via Nutrition Risk Screening 2002: Insights from a National Cohort

**DOI:** 10.3390/nu17162716

**Published:** 2025-08-21

**Authors:** Nadir Yalçın, Merve Kaşıkcı, Burcu Kelleci-Çakır, Kutay Demirkan, Karel Allegaert, Meltem Halil, Mutlu Doğanay, Osman Abbasoğlu

**Affiliations:** 1 Department of Clinical Pharmacy, Faculty of Pharmacy, Hacettepe University, Ankara 06100, Türkiye; burcukey@yahoo.com (B.K.-Ç.); kutay@hacettepe.edu.tr (K.D.); 2 Department of Biostatistics, Faculty of Medicine, Hacettepe University, Ankara 06100, Türkiye; mervekasikci@outlook.com; 3 Department of Pharmaceutical and Pharmacological Sciences, KU Leuven, 3000 Leuven, Belgium; karel.allegaert@kuleuven.be; 4 Department of Development and Regeneration, KU Leuven, 3000 Leuven, Belgium; 5 Department of Hospital Pharmacy, Erasmus University Medical Center, 3015GD Rotterdam, The Netherlands; 6 Department of Internal Medicine, Division of Geriatrics, Faculty of Medicine, Hacettepe University, Ankara 06100, Türkiye; meltemhalil@yahoo.com; 7 Department of General Surgery, Gulhane Medical Faculty, Bilkent City Hospital, University of Health Sciences, Ankara 06010, Türkiye; drmdoganay@gmail.com; 8 Clinical Nutrition Master’s Program, Hacettepe University, Ankara 06100, Türkiye; osmanabbasoglu@yahoo.com

**Keywords:** malnutrition, inpatient, artificial intelligence, nutritional support team, primary care

## Abstract

*Purpose:* This study aimed to develop and validate a new machine learning (ML)-based screening tool for a two-step prediction of the need for and type of nutritional therapy (enteral, parenteral, or combined) using Nutrition Risk Screening 2002 (NRS-2002) and other demographic parameters from the Optimal Nutrition Care for All (ONCA) national cohort data. *Methods:* This multicenter retrospective cohort study included 191,028 patients, with data on age, gender, body mass index (BMI), NRS-2002 score, presence of cancer, and hospital unit type. In the first step, classification models estimated whether patients required nutritional therapy, while the second step predicted the type of therapy. The dataset was divided into 60% training, 20% validation, and 20% test sets. Random Forest (RF), Artificial Neural Network (ANN), deep learning (DL), Elastic Net (EN), and Naive Bayes (NB) algorithms were used for classification. Performance was evaluated using AUC, accuracy, balanced accuracy, MCC, sensitivity, specificity, PPV, NPV, and F_1_-score. *Results:* Of the patients, 54.6% were male, 9.2% had cancer, and 49.9% were hospitalized in internal medicine units. According to NRS-2002, 11.6% were at risk of malnutrition (≥3 points). The DL algorithm performed best in both classification steps. The top three variables for determining the need for nutritional therapy were severe illness, reduced dietary intake in the last week, and mild impaired nutritional status (AUC = 0.933). For determining the type of nutritional therapy, the most important variables were severe illness, severely impaired nutritional status, and ICU admission (AUC = 0.741). Adding gender, cancer status, and ward type to NRS-2002 improved AUC by 0.6% and 3.27% for steps 1 and 2, respectively. *Conclusions:* Incorporating gender, cancer status, and ward type into the widely used and validated NRS-2002 led to the development of a new scale that accurately classifies nutritional therapy type. This ML-enhanced model has the potential to be integrated into clinical workflows as a decision support system to guide nutritional therapy, although further external validation with larger multinational cohorts is needed.

## 1. Introduction

Malnutrition affects populations significantly (30–40%) both in daily life and during illnesses, leading to impaired quality of life and increased complications and mortality rates [1,2]. Malnutrition can be caused by social and economic factors, such as poverty, hunger, and inequalities in reaching food, and also may be related to an underlying disease. Policies and strategies to fight against malnutrition at national and international levels are required to improve patient care. Identifying patients who are at risk of malnutrition is crucial for the adequate management of patient care [3,4].

Routine nutritional screening is recommended in all healthcare settings as part of the Optimal Nutrition Care for All (ONCA) campaign, a multi-stakeholder initiative led by the European Nutritional Health Alliance (ENHA) to facilitate improved screening for disease-related risk of malnutrition and to promote the implementation of nutrition care at the national level across Europe [5,6]. It is known that disease-related malnutrition burdens the European Union budget by EUR 171 billion per year. Therefore, after the European Parliament declared in October 2008 that malnutrition is among the main priorities of the European Union Health Strategy, ENHA carried out the campaign “Fight against malnutrition” between 2008 and 2013 [7].

Several nutritional screening tools for malnutrition have been developed and validated in different populations and countries [1,8]. Among these, the Nutritional Risk Screening (NRS-2002) has become particularly well established and has been commonly used worldwide [2]. NRS-2002 is composed of two sections: the initial screening consists of four “Yes/No” questions [low body mass index (BMI) (<20.5 kg/m^2^), weight loss during the last 3 months, reduced dietary intake during the last week, and presence of severe illness], and if at least one question is answered with “Yes”, the main screening proceeds. One score is added if the patient is ≥70 years old, leading to a maximum score of 7. A total score of ≥3 is regarded as being at risk of malnutrition [9]. NRS-2002 assesses disease severity, taking into account the stress metabolism induced by the degree of the illness [2,8,10]. Even though some other tools are limited to certain patient groups, the validity of NRS-2002 has been shown in both inpatient and outpatient settings in primary, secondary, and tertiary hospitals [4,11].

To the best of our knowledge, there is no study about machine learning (ML)-focused precision nutritional therapy and evaluation of other prognostic factors of malnutrition risk in large cohorts. Our primary objective was to develop and validate an ML-based model that predicts the need for nutritional therapy with NRS-2002 and additional demographic parameters obtained from ONCA national cohort data. The secondary objective was to develop and validate an ML-based model that predicts the type of nutritional therapy (enteral vs. parenteral vs. combined therapy) using the associated parameters. Therefore, this research can be considered a hypothesis-testing study aimed at developing ML-based models that integrate demographic information with NRS-2002 to predict the need for and type of nutritional therapy. The models developed in this study are intended for clinical implementation for supporting clinicians in their decision-making process.

## 2. Methods

### 2.1. Data Collection

**Figure 1:**
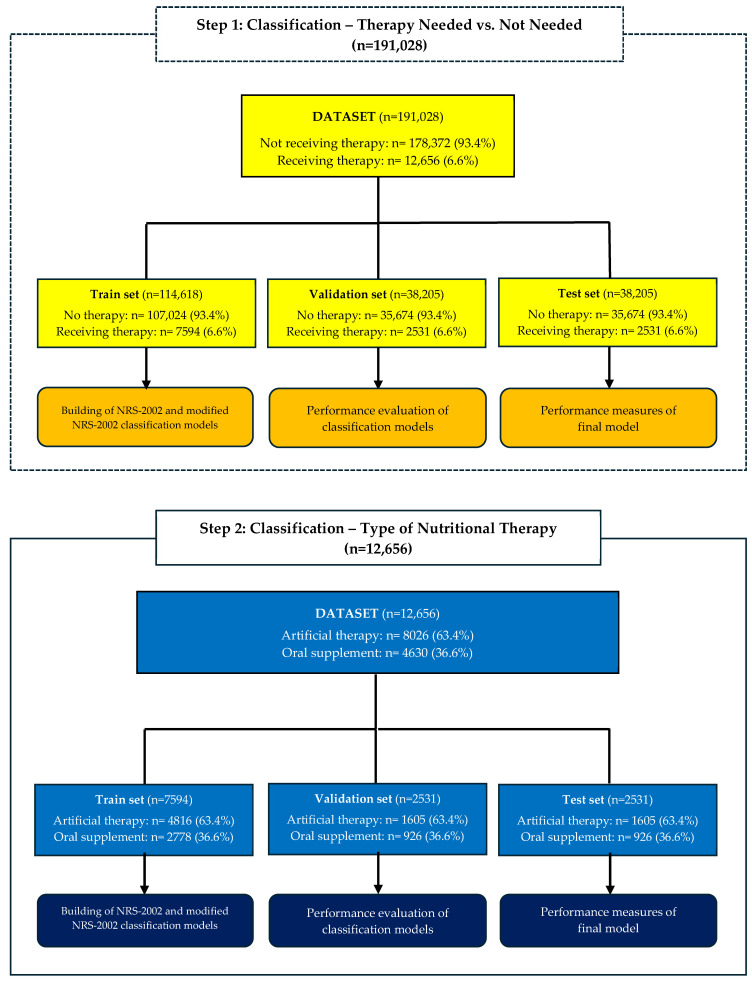
Flow chart of machine learning procedure.

Multicenter national data were provided from Türkiye, which is among the focus countries of ONCA, a multi-stakeholder initiative launched in 2014. The ultimate goal of the campaign is to screen the nutritional status of all patients hospitalized in Türkiye, to identify those at risk of disease-related malnutrition, and to provide appropriate treatment. The data obtained include information on each patient’s age, gender, BMI, NRS-2002 results, presence of cancer, and the admitted clinic. All data are based on actual measurements (BMI and NRS-2002) determined by the clinician. This study consists of two steps. The first step focuses on determining if the patients receive nutritional therapy, while the second step focuses on identifying the type of nutritional therapy (Figure 1).

### 2.2. Statistical Analysis

Analyses were performed by an academic statistician using the free and open-source language R (software version 4.4.1, http://www.rproject.org). Analysis was conducted on the 64-bit Windows operating system. Continuous variables were summarized as mean (standard deviation, SD) and categorical variables were summarized as frequency (percentage, %). All the variables were treated as categorical. Numerical variables were also categorized according to clinically established NRS-2002 thresholds to maintain interpretability and comparability with previous studies. Although this approach may reduce granularity, it was considered essential for clinical relevance. Given the absence of numerical variables, no issues related to outliers were observed. No missing values were handled during preprocessing prior to this analysis. Although the number of observations is high, the number of variables is limited (*p* = 10). The hospital information management system records, completed during patient admission, include mandatory questions required by the Ministry of Health, which must be answered by the clinician. In addition to the mandatory fields ensuring completeness, data quality was further assured through standardized data entry procedures across centers, routine plausibility checks by clinicians, and consistency controls embedded in the hospital information management system. Within the scope of the ONCA project, patient registration cannot be finalized unless all required fields are completed. Consequently, no imputation was necessary. Also, the size of the dataset was considered sufficient for the ML model. No resampling or augmentation techniques, such as down-sampling or up-sampling, were applied to the dataset. The statistical significance and effects size of the variables were examined individually by using univariate analysis. Pearson chi-squared tests were used for categorical variables. The statistical significance (α) was taken as 0.05. Since *p*-value is affected by the sample size, effect sizes (Phi coefficient for 2 × 2 tables, Cramer’s V coefficient for rxc tables) are also given. According to Cohen [12], effect sizes of 0.10, 0.30, and 0.50 correspond to small, medium, and large effects, respectively. Pearson chi-squared tests were conducted by using stats R package [13], and effect sizes were calculated by using rcompanian R package [14]. The importance of variables was assessed using SHapley Additive exPlanations (SHAP) values with the h2o and shapviz R packages [15,16]. ggplot2 [17] and gridExtra [18] R packages were used to arrange and combine the plots. SHAP is a method used to explain the decision-making processes of machine learning models by quantitatively evaluating the contribution of each variable to the model’s predictions. SHAP values indicate both the direction (positive or negative) and magnitude of a variable’s impact on the model output, thereby enhancing the interpretability of the decision-making process. This, in turn, improves the transparency of nutritional therapy management predictions and strengthens the model’s reliability in clinical applications. Since the number of observations significantly exceeded the number of features, feature selection was not performed. However, the importance of the features in the model was evaluated using SHapley Additive exPlanations (SHAP) values. Additionally, partial dependence plots were generated by using the h2o.partialPlot function in h2o R package to enhance model interpretability. These plots provide a graphical representation of the marginal effects of variables on the classification probability. In our study, we utilized the Checklist for Ethical and Effective Application of AI/ML Modeling in Nutrition Research [19].

### 2.3. Classification Analysis

Supervised machine learning methods were used in this study because the aim was to develop classification models. Currently, the models have not been updated with additional data. Since the dataset used in this study is large in terms of the number of observations, ML models were preferred over classical statistical analysis methods due to their ability to efficiently process large amounts of data and model both linear and nonlinear relationships between variables. Due to the large number of observations and the preservation of prevalence in the data, no class balancing was applied. Therefore, when reporting performance metrics, metrics recommended for imbalanced class distributions (such as balanced accuracy and MCC) are specifically provided. Classification models were developed to estimate whether or not patients received nutritional therapy in the first step, as well as the type of nutritional therapy they received in the second step. For both steps, models were obtained based on two different variable sets. The first set of variables was referred to as NRS-2002 and the second set as AI-modified NRS-2002.

For classification analyses, the dataset was divided into 60% train, 20% validation, and 20% test set. There are several methods, such as hold-out, cross-validation, and bootstrap sampling, to ensure the validity of ML models. In this study, various classification algorithms were employed to examine their predictive performance. Since the number of observations is large, parameter optimization for each algorithm is time-consuming. Due to the computational power and analysis time required, and considering the large size of the dataset, the hold-out method was preferred over cross-validation and bootstrap. The hold-out method used in this study is referred to as three-way hold-out [20,21]. This is because the dataset was divided into three sets: train, validation, and test. The model with the best performance was evaluated on an independent test set, which was randomly selected from the same cohort and constitutes 20% of the data. Nevertheless, the results were not validated with data from a different center. The flow chart is provided to summarize the number of observations in each set. Random Forest (RF), Artificial Neural Network (ANN), deep learning (DL), Elastic Net Regularized Generalized Linear Model (EN), and Naive Bayes (NB) algorithms were used for classification. The classification methods were selected from among the methods that are both commonly used in the literature and supported by the R package employed in this study. Models were developed using the train set. The grid search procedure was employed to optimize the hyperparameters. Grid search is the process of selecting the most appropriate value within the specified value range of certain hyperparameters for the algorithms of interest. The number of trees and maximum tree depth for RF, the number of neurons for ANN, the number of hidden layers, the number of neurons, and the epoch number for DL, lambda value for EN, and Laplace value for NB were optimized. To ensure the reproducibility of the analysis, the seed number was set to 123. The codes used in the classification were uploaded to this GitHub repo: https://github.com/mervekasikci/ONCACohortStudy (accessed on 3 March 2025).

The validation set was used for the comparison of model performances and model selection. Area under the ROC curve (AUC), accuracy, balanced accuracy, Matthew’s correlation coefficient (MCC), sensitivity, specificity, positive predictive value (PPV), negative predictive value (NPV), and F1 score were used as performance measures. Among these metrics, AUC has an accepted threshold in the literature. An AUC value of at least 0.70 is required for the model to have a good level of discriminative ability [22]. To enable a detailed examination of the models’ performance in classifying positive and negative classes, sensitivity, specificity, positive predictive value, and negative predictive value were provided. For the evaluation of the overall performance of models, accuracy and F1 score were reported. Since there was class imbalance in the models (especially in step 1), the Matthews correlation coefficient (MCC) and balanced accuracy, which are recommended for such cases, were specifically included in the results. Classification analyses were conducted using the h2o package [16], performance measures were calculated using the GMDH2 package [23], and AUC values were obtained using the pROC package [24].

## 3. Results

### 3.1. Descriptive Statistics and Univariate Analysis

A total of 191,028 patients were included in this study. Of these, 8.1% had a BMI < 20.5, 13.6% had weight loss within 3 months, 16.2% had reduced dietary intake in the last week, 31.4% had severe illness, and 2.1% had severely impaired nutritional status. According to NRS-2002, 11.6% of the patients were at malnutrition risk (≥3 points). Of the patients, 54.6% were male, 9.2% had cancer, and 49.9% were hospitalized in internal medicine units. Descriptive statistics for the entire dataset are given in Table 1. In Table 2, the distribution of variables is presented according to whether the patients received nutritional therapy or not, and in Table 3 the distribution of variables is presented according to the type of nutritional therapy.

### 3.2. Variable Importance

**Figure 2:**
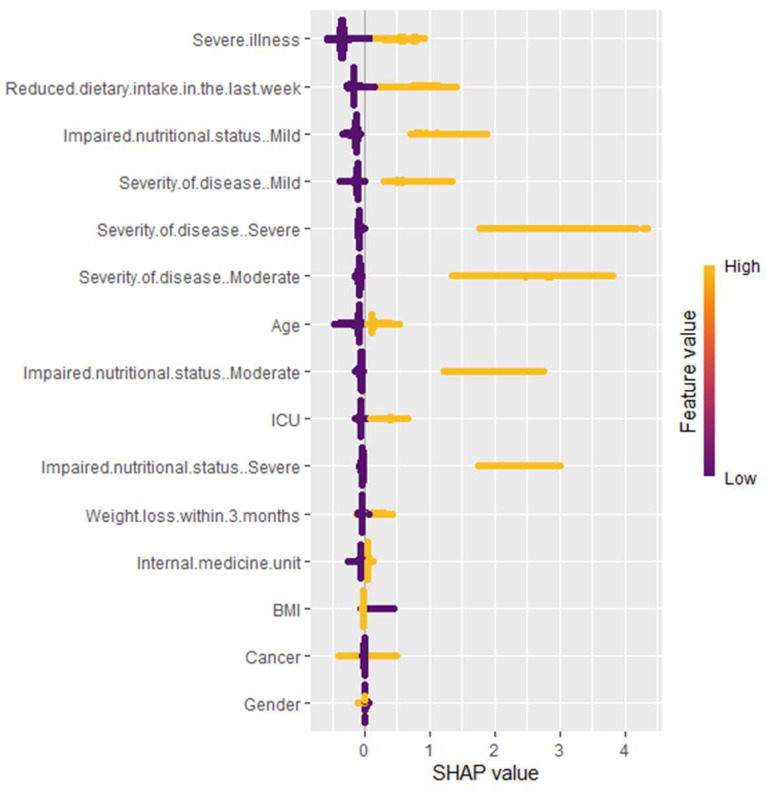
Variable importance plot based on SHAP values for step 1. This SHAP summary plot illustrates the impact of various clinical and nutritional factors on the model’s predictions. Each point represents an individual patient’s data, with color indicating feature values (yellow = high, purple = low). The *x*-axis represents SHAP values, which measure the contribution of each feature to the prediction. A higher SHAP value suggests a stronger positive influence on the outcome, while lower values indicate a negative or neutral effect.

**Figure 3:**
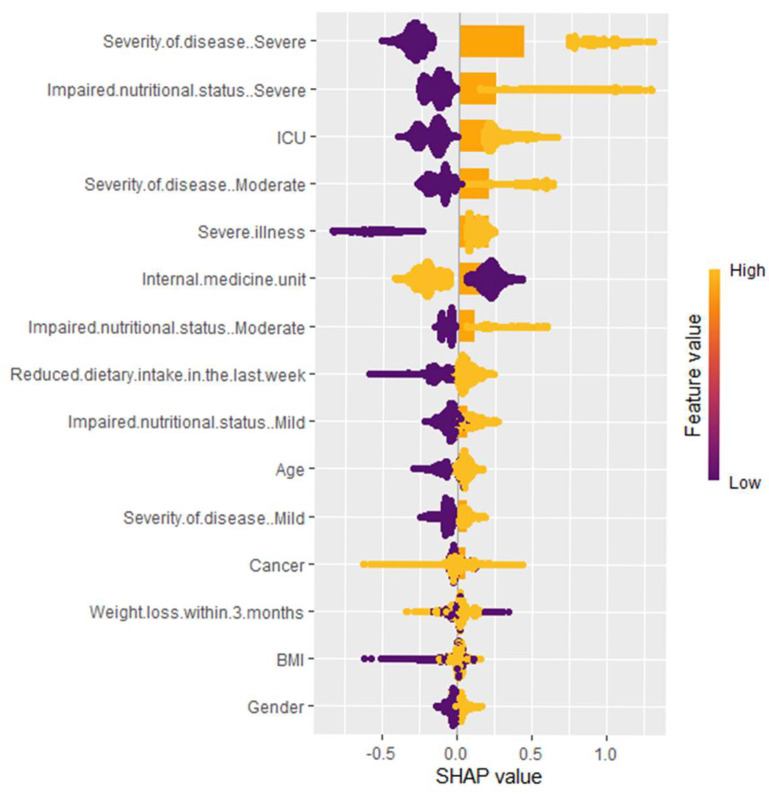
Variable importance plot based on SHAP values for step 2. This SHAP summary plot illustrates the impact of various clinical and nutritional factors on the model’s predictions. Each point represents an individual patient’s data, with color indicating feature values (yellow = high, purple = low). The *x*-axis represents SHAP values, which measure the contribution of each feature to the prediction. A higher SHAP value suggests a stronger positive influence on the outcome, while lower values indicate a negative or neutral effect.

Since the SHAP method is part of the explainable ML approach, it overcomes the black box problem in ML methods and improves the interpretability of the model. The variable importance rankings based on the SHAP method for classification are summarized in Figure 2 and Figure 3. The contributions of variables to distinguishing receiving nutritional therapy versus not receiving nutritional therapy are shown in Figure 2. The variable contributions to determining the type of nutritional therapy are shown in Figure 3. The three most important variables for step 1 in determining the need for nutritional therapy were severe illness, reduced dietary intake in the last week, and mild impaired nutritional status. The three most important variables for step 2 in determining the type of nutritional therapy were severe illness, severely impaired nutritional status, and ICU admission.

Key insights for clinicians in step 1 (Figure 2):

- Severe illness and reduced dietary intake in the last week have a wide range of SHAP values (yellow and purple), showing that high and low values greatly impact the predictions of the model.
- Impaired nutritional status (mild and moderate), severity of disease (mild, moderate, and severe), age, ICU, and weight loss within 3 months show a medium range of SHAP values. This means that their impact on the model’s prediction varies moderately with different values, but overall, they are not as strong in influence compared to other top variables.
- Cancer and gender display a narrow range of SHAP values, indicating that while they contribute to the predictions, their impact is relatively stable and less variable.

Key insights for clinicians in step 2 (Figure 3):

- Severity of disease (particularly severe cases), ICU admission, and impaired nutritional status are the most influential factors. Both high and low categories of these variables may greatly impact the model’s predictions.
- Severe illness and internal medicine unit admission also contribute substantially, highlighting the importance of hospitalization context.
- Reduced dietary intake in the last week indicates that larger reductions in intake are associated with higher model predictions, while smaller reductions result in lower predictions.
- Age, cancer status, and weight loss within 3 months, as well as moderate illness severity and mild to moderate impaired nutritional status, have a medium range of SHAP scores. Their impact on the model’s predictions is noticeable, although not as strong as the top variables.
- BMI and gender have little effect on model predictions.

### 3.3. Classification Analysis

The variables included in these sets are presented in Table 4. Dummy variable coding was performed for variables with more than two categories.

The validation set performances of classification models are given in Table 5 and Table 6 for step 1 and step 2, respectively. Using these tables, model performances were evaluated. For step 1, the DL model with the modified NRS-2002 variable set had higher classification performances in terms of AUC, MCC, balanced accuracy, and F1 score. The AUC values of NRS-2002 and modified NRS-2002 models of the DL algorithm were compared, and the difference between them was found to be statistically significant (Z = −5.2, *p* < 0.0001). Therefore, among the models presented in Table 5, the DL model with the modified NRS-2002 variable set was selected as the best model. This model contains 10 hidden layers, 50 neurons in each layer, and 10 epochs. In addition, the performance of the modified NRS-2002 model of the EN algorithm was close to the DL model with the modified NRS-2002 variable set.

For step 2, the results of the validation set are similar to step 1 in terms of model comparison. The classification performances of the DL model with the modified NRS-2002 variable set were greater than other models in terms of AUC, MCC, balanced accuracy, and F1 score. The AUC values of the NRS-2002 and modified NRS-2002 models of the DL algorithm were compared, and the difference between them was significantly different (Z = −3.83, *p* = 0.0001). Consequently, the DL model with the modified NRS-2002 variable set was chosen as the optimal model from the models listed in Table 6 for step 2. This model contains 2 hidden layers, 50 neurons in each layer, and 10 epochs.

**Figure 4:**
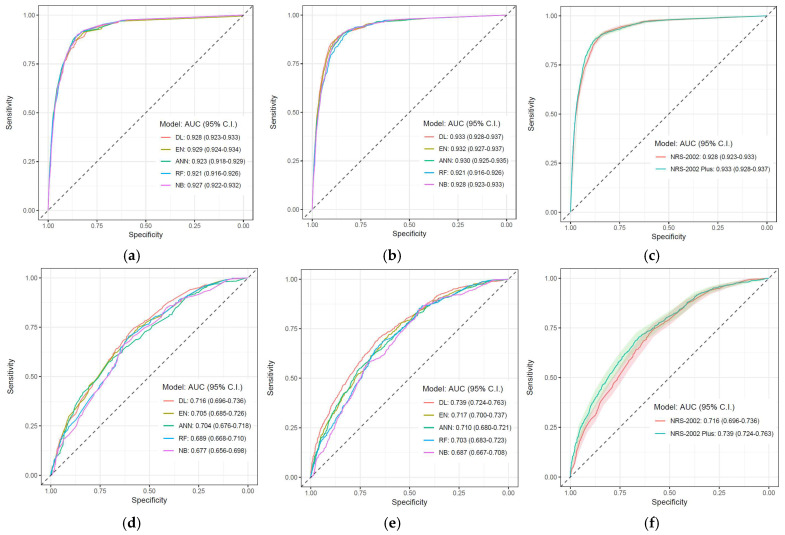
AUC ROC curves of the models. (**a**,**b**) compare the performance of the models of NRS-2002 and AI-based modified NRS-2002 for step 1, respectively, while (**c**) compares only the model performances of the selected DL. (**d**,**e**) compare the performance of the models of NRS-2002 and AI-based modified NRS-2002 for step 2, respectively, while (**f**) compares only the model performances of the selected DL.

The DL algorithm with the modified NRS-2002 variable set performed well in terms of classification in both steps. The performance of the DL algorithm stands out in step 2 when compared to other algorithms. While the NRS-2002 variable set includes six variables, the modified NRS-2002 model includes three additional variables (gender, cancer, and unit). Table 7 illustrates the absolute and relative differences in the performance metrics of the DL algorithm between the NRS-2002 and modified NRS-2002 models. Also, Figure 4 visualizes the AUC curves that best explain the predictive performance of a model for both step 1 and step 2.

The performance of the selected model (DL algorithm with modified NRS-2002 variable set) was further tested on an independent test set. The test set performances for both steps are provided in Table 8. According to the results, the performance of the validation and test sets are similar for both steps.

**Figure 5:**
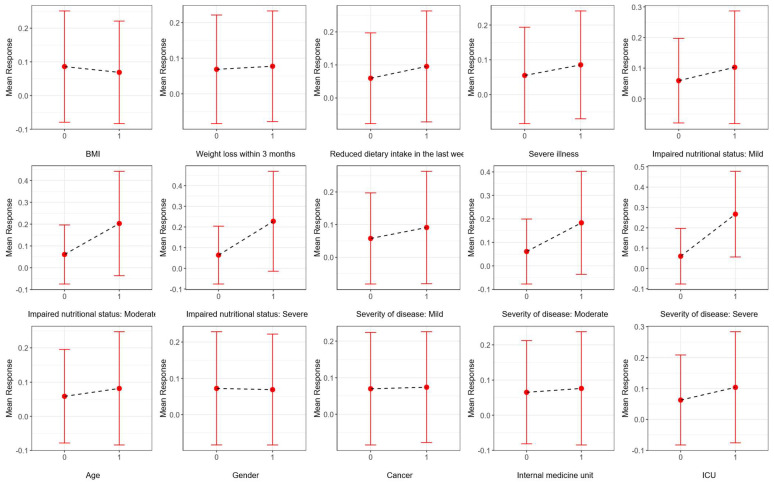
Partial dependence plots, illustrating the influence of each input variable on the predictions generated by the AI-based modified NRS-2002 model for step 1.

**Figure 6:**
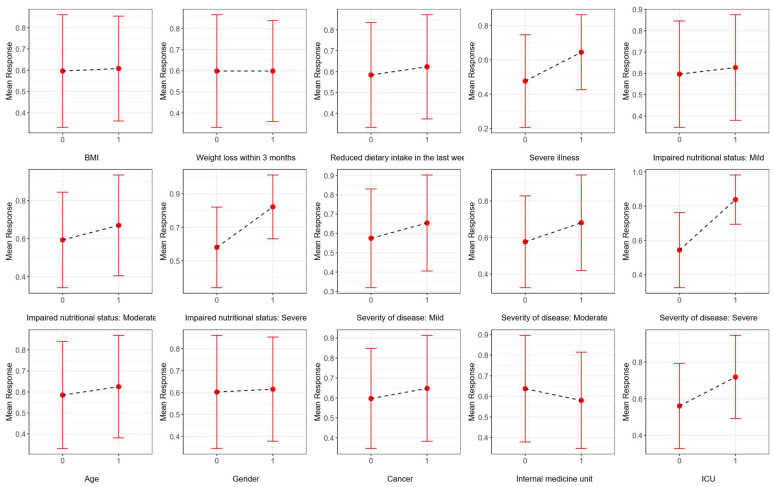
Partial dependence plots, illustrating the influence of each input variable on the predictions generated by the AI-based modified NRS-2002 model for step 2.

In order to enhance the explainability of the selected model, partial dependence plots are provided using a test set. Since all the input variables used in this study are categorical, the *x*-axis displays the categories of these variables. The *y*-axis presents the mean response—defined as the probability of the positive class—along with the corresponding standard deviations. Figure 5 illustrates the marginal effects of the input variables in step 1 of the AI-based modified NRS-2002 model, while Figure 6 illustrates their effects in step 2. The marginal effect of each variable is evaluated based on the change in the mean response when the variable changes from one category to another. According to Figure 5, the model’s prediction of the positive class increases slightly when BMI category is <20.5 compared to ≥20.5 in step 1. However, when the “severity of the disease” category is severe or the “impaired nutritional status” is severe, the model’s prediction for the positive class has increased noticeably. This indicates that, while BMI has a small contribution to the model’s outcome, the severity of the disease and impaired nutritional status have a greater impact on the classification decisions of the model. The effects of these two input variables also influence the positive class prediction in step 2.

## 4. Discussion

Given the negative impact of malnutrition on nutritional therapy, prognosis, and economic burden, mandatory screening and assessment are conducted in many clinical and healthcare settings. Nutritional risk screening facilitates the early identification of malnutrition, requiring tools that are concise, affordable, and effective [25]. This study is of value as it demonstrates the power of NRS-2002 with artificial intelligence in predicting malnutrition therapy and type of nutritional therapy and the aspects that can be improved.

Following the initial publication of the NRS-2002, it was incorporated into the ESPEN guidelines, where it was indicated that it could be used as a standalone screening tool in hospitals or, when necessary, in conjunction with other population-specific screening tools [26]. Compared to other tools, it offers several advantages, including ease of use, speed, high reproducibility, and efficiency. Unlike other methods, it also assesses recently consumed foods, allowing for risk evaluation based on decreased appetite. Studies comparing NRS-2002 with other nutritional screening tools have demonstrated that this method has high specificity and sensitivity in patients with different clinical conditions and ages. It also offers greater accuracy and predictive power in terms of clinical assessment, mortality, and length of hospital stay [2,27,28]. All these features show that NRS-2002 still appeals to the majority of the population, even after many years. This statement is supported by the fact that NRS-2002 gives very strong results in the DL model in a very large population in this study.

Although NRS-2002 is recognized as an important screening tool for the assessment of nutritional status in clinical settings, there are areas where this tool could be further improved [29]. Therefore, the inclusion of some additional variables in the screening test may improve the decision-making process of nutritional therapy. In this study, we demonstrated in a large cohort how NRS-2002 with additional variables can best predict whether patients will receive nutritional therapy and, if so, the type of therapy with the support of artificial intelligence. Previous reports showed that 20–45% of the patients had malnutrition upon hospitalization [2]. In Barbosa et al.’s study, 46.4% of the patients were at risk of malnutrition [27]. In our study, 11.6% of the patients were at risk of malnutrition. Although the population does not appear to be at risk for malnutrition, the NRS-2002 screening following hospitalization was large enough for further analysis. The primary reasons for the lower number of malnourished patients compared to studies analyzing the prevalence of malnutrition in hospitalized patients are presumed to be differences in the study population, limitations of the screening method used, and the impact of clinical interventions.

When the most important clinical parameters for machine learning were examined, the three most important parameters for deciding whether to receive nutritional therapy or not were determined as “severe illness, decreased dietary intake in the last week, and mild impaired nutritional status”. Subsequently, the parameters “severe disease status, severely impaired nutritional status, and ICU admission” were determined in deciding the type of nutritional therapy. Since these important parameters were also stated as important in the initial development of NRS-2002 [26], they prove that the machine learning models developed are working correctly. It has been reported that gender, one of the prominent variables for the new version of NRS-2002, is important in terms of nutritional risk. In Barbosa’s study (763 patients, of whom the majority were men (50.5%)), men were 2.04 times more likely to present nutritional risk than women [27]. Assessments that take into account the gender factor can provide more specific results based on the metabolic differences in individuals. In this study, it was proved that the power of the model increased with the addition of the gender factor to the screening tool. According to a multicenter randomized study, it has been demonstrated that female hospitalized patients with multimorbidity exhibit a different clinical presentation and are more prone to loss of appetite and a decrease in daily dietary intake compared to male hospitalized patients. In our study, gender was identified as one of the most significant variables at both stages, and when included in NRS-2002, a significant improvement in model prediction performance was observed [30].

Another parameter was cancer status. While other chronic comorbidities may also contribute to malnutrition risk, cancer was selected as a representative morbidity due to its high prevalence and strong prognostic implications in hospitalized populations. Although NRS-2002 has not yet reached the ability to classify malnutrition based on indicators such as reduced muscle mass, low body mass index, and weight loss, it is the optimal and superior initial nutritional risk screening tool rather than Malnutrition Screening Tool, Short-Form of Mini-Nutritional Assessment, and MUST in the Global Leadership Initiative on Malnutrition (GLIM) process to detect malnutrition in relation to survival [31,32]. In a study conducted with oncology patients, NRS-2002 showed better correlation with GLIM criteria among adults with cancer and was reported to be a good candidate for first-step malnutrition risk screening according to the GLIM diagnostic scheme [25]. The presence of chronic diseases, such as cancer, can significantly affect nutritional requirements. It is currently shown in the developed model that cancer status should be considered as an item in its own right when assessing serious illness status in NRS-2002.

The last parameter recommended to be added to the model is the ward in which the patient is located. The nutritional requirements of a patient in an intensive care unit are quite different from those of a patient in a surgical ward [33]. Although the NRS-2002 is well known to have the highest sensitivity among elderly patients admitted to ICUs when compared with the Mini Nutrition Assessment, there is no current literature specifically comparing surgical, internal medicine, and ICU admissions [34]. Taking ward-specific differences into account can make nutrition therapy decisions more targeted and individualized [6]. Likewise, in the screening tool currently in use, whether the patient is in intensive care conditions is assessed in the first step. In the new model, it was stated that the model provided a better prediction when the surgical unit, internal medicine unit, and ICU were questioned in the second part.

While proposals to enrich NRS-2002 aim to provide more precise and individualized approaches to clinical decision making, artificial intelligence and especially deep learning methods have shown significant potential in this field in recent years [35]. Compared to traditional statistical methods, deep learning stands out with its capacity to analyze large datasets and reveal complex relationships, as in this study [35,36]. This makes it possible to integrate multidimensional data (gender, presence of cancer, inpatient service, laboratory results, etc.) in nutritional status assessment and make more accurate predictions. This study stands out as one of the rare studies in the literature comparing different artificial intelligence models in predicting whether patients will receive nutritional therapy (step 1) and the type of nutritional therapy by working with a very large dataset. Among the models tested (RF, ANN, DL, EN, and NB), DL was found to be especially prominent according to AUC and F1 scores. Looking at the difference between the NRS-2002 and modified NRS-2002 models for step 1, it is shown that modified NRS-2002 has an increase of 0.6% in AUC, while for step 2 it has an increase of 3.27%. Sensitivity and specificity plots also show that DL performs better in different models.

According to the test set’s results, the false-positive ratios of the proposed model (DL algorithm with modified NRS-2002 variable set) are 0.281 and 0.206, respectively. However, the false positive ratio was increased from 0.063 in step 1 to 0.431 in step 2. This can be attributed to the reduction in the number of observations in step 2 compared to step 1, as well as the aim to distinguish more homogeneous classes. The proposed model for step 1 misses 28.1% of individuals who actually need support. For step 2, the model misses 20.6% of those receiving enteral/combined/parenteral nutritional therapy. Given that clinical responses may change over time, potential harm and risks can be minimized by using the model routinely on a daily or weekly basis. The proposed models are not intended to be the primary decision maker but rather an additional support system designed to assist clinicians.

Beyond the predictive performance of the modified NRS-2002, it is essential to consider how this AI-enhanced screening tool can be effectively integrated into existing clinical workflows. The observed false-positive rates (28.1% in step 1 and 43.1% in step 2) highlight the potential for increased clinical burden, unnecessary consultations, and additional resource allocation if not carefully managed. Thus, future implementations should emphasize workflow optimization and cost–benefit analyses to ensure that the added predictive capacity translates into tangible clinical value. Moreover, experiences from other AI-driven clinical decision support systems suggest that integration with electronic health records, automated alerts, and multidisciplinary nutrition teams can facilitate more seamless adoption and reduce clinician workload [6,35]. Comparisons with contemporary AI-enabled applications in oncology and critical care nutrition have also shown promising results in risk stratification and individualized nutritional planning [31,32]. Therefore, contextualizing the proposed model within these broader developments will strengthen its clinical relevance and provide insights for its refinement and external validation.

The strength of this study is that, since it is a multicenter national cohort study, the large data pool obtained can be effective in the modified NRS-2002. While the accuracy of the nutritional database has been enhanced, the gains are modest, which may limit its practical clinical impact. However, its implementation in health systems could have a relevant clinical impact, especially in critical and geriatric care. The database’s origin in a single country is another factor influencing its clinical applicability, but it paves the way for the incorporation of other international databases. The actual relevance of the proposed procedure can be determined through a comparison of these databases. Among the limitations, it has not yet been determined in which section of NRS-2002 the new parameters identified will be integrated. In our study, the addition of gender, cancer status, and ward type significantly improved predictive performance, yet the specific method of integration into the NRS-2002 scoring system remains undefined. At present, these findings should be regarded as hypothesis-generating rather than a finalized scoring proposal. Future work should focus on establishing appropriate weighting methods (e.g., expert consensus, Delphi panels, or regression-based coefficients) to incorporate these variables into NRS-2002 in a standardized and clinically applicable manner. Such methodological refinement will be essential before clinical implementation can be recommended. It is thought that in the future, the scores of the new parameters and the section in which they should be included will be determined and the enriched NRS-2002 can be easily used in clinical practice. A key limitation to acknowledge is that the modified NRS-2002 requires rigorous validation across diverse populations prior to its widespread implementation. Also, our study did not assess clinical outcomes such as length of stay, mortality, or complications; future research should validate the predictive performance of the model against these endpoints. In addition to these limitations, it should be underlined that our findings are based exclusively on data collected from medical institutions in Türkiye. Therefore, the generalizability of the developed model to other countries, different ethnic groups, and healthcare systems remains to be established. Future research should aim at multinational and multi-institutional external validation studies, which would allow assessment of robustness across diverse clinical environments. Moreover, integration into clinical practice (e.g., web-tool, mobile application) will require careful evaluation of feasibility, cost-effectiveness, and workflow optimization to ensure that the added predictive value translates into tangible clinical benefits. At the same time, the lack of data such as muscle mass, protein and caloric goals, nutritional therapy duration, and hospitalization period prevented us from developing the model more precisely.

Furthermore, no sampling techniques were applied despite class imbalance, as the large sample size preserved prevalence rates and allowed for robust evaluation with balanced accuracy and MCC. Future studies may investigate the integration of resampling methods or cost-sensitive learning to further optimize model performance, as well as conduct external validation with larger multinational cohorts.

## 5. Conclusions

In this study, it was shown that an NRS-2002 enriched with additions (gender, cancer, and type of ward) could not only more accurately determine whether nutritional therapy is needed but also help to make more accurate decisions about the type of nutrition (enteral, parenteral, or combined) after initiation of therapy. NRS-2002 is an important guideline for the initiation of nutritional therapy in patients and the determination of the method (artificial nutritional therapy vs. oral nutritional supplement). It has been shown that this situation will be further strengthened with the addition of the three variables studied. One of the most important advantages of DL algorithms is that the model can continuously improve itself in terms of high-performance prediction by being trained with new data. However, to achieve this, there is a need for new clinical studies developed using existing GitHub code, based on data obtained from different centers, preferably on an annual basis. Over time, this will allow for further optimization of clinical outcomes and the most appropriate nutritional therapy plans for patients. An NRS-2002 integrated with DL could provide clinicians with more reliable and evidence-based decision support systems. In the future, larger multinational/multicenter studies with heterogeneous data are needed to demonstrate the predictive performance of the developed and validated modified NRS-2002.

## Figures and Tables

**Table 1:** Descriptive statistics of the dataset.

	Frequency (n)	Percent (%)
**BMI**		
<20.50	15,389	8.1
≥20.50	175,639	91.9
Total	191,028	100.0
**Weight loss within 3 months**		
Present	25,904	13.6
Absent	165,124	86.4
Total	191,028	100.0
**Reduced dietary intake in the last week**		
Present	31,016	16.2
Absent	160,012	83.8
Total	191,028	100.0
**Severe illness**		
Present	60,013	31.4
Absent	131,015	68.6
Total	191,028	100.0
**Impaired nutritional status**		
Absent	158,508	82.9
Mild	22,502	11.8
Moderate	6094	3.2
Severe	3924	2.1
Total	191,028	100.0
**Severity of disease**		
Absent	140,618	73.6
Mild	36,325	19.0
Moderate	7447	3.9
Severe	6638	3.5
Total	191,028	100.0
**Age**		
<70	116,226	60.8
≥70	74,802	39.2
Total	191,028	100.0
**Gender**		
Male	104,219	54.6
Female	86,809	45.4
Total	191,028	100.0
**Cancer**		
Absent	173,378	90.8
Present	17,650	9.2
Total	191,028	100.0
**Unit**		
Internal medicine	95,364	49.9
Surgery	67,398	35.3
Intensive care	28,266	14.8
Total	191,028	100.0

**Table 2:** Descriptive statistics and univariate analysis results regarding whether patients received nutritional therapy or not.

	Not Receiving Nutritional Therapy (n = 178,372, 93.4%)		Receiving Nutritional Therapy (n = 12,656, 6.6%)			
	Frequency (n)	Percent (%)	Frequency (n)	Percent (%)	*p*-Value	Effect Size
**BMI**						
<20.50 (n = 15,389, 8.1%)	12,578	81.7	2811	18.3	<0.001	0.139
≥20.50 (n = 175,639, 91.9%)	165,794	94.4	9845	5.6		
**Weight loss within 3 months**						
Present (n = 25,904, 13.6%)	19,546	75.5	6358	24.5	<0.001	0.285
Absent (n = 165,124, 86.4%)	158,826	96.2	6298	3.8		
**Reduced dietary intake in the last week**						
Present (n = 31,016, 16.2%)	23,154	74.7	7862	25.3	<0.001	0.331
Absent (n = 160,012, 83.8%)	155,218	97.0	4794	3.0		
**Severe illness**						
Present (n = 60,013, 31.4%)	49,682	82.8	10,331	17.2	<0.001	0.288
Absent (n = 131,015, 68.6%)	128,690	98.2	2325	1.8		
**Impaired nutritional status**						
Absent (n = 158,508, 83%)	155,352	98.0	3156	2.0	<0.001	0.452
Mild (n = 22,502, 11.8%)	17,576	78.1	4926	21.9		
Moderate (n = 6094, 3.2%)	3521	57.8	2573	42.2		
Severe (n = 3924, 2.1%)	1923	49.0	2001	51.0		
**Severity of disease**						
Absent (n = 140,618, 73.6%)	138,861	98.8	1757	1.2	<0.001	0.457
Mild (n = 36,325, 19%)	31,515	86.8	4810	13.2		
Moderate (n = 7447, 3.9%)	4488	60.3	2959	39.7		
Severe (n = 6638, 3.5%)	3508	52.8	3130	47.2		
**Age**						
<70 (n = 116,226, 60.8%)	111,810	96.2	4416	3.8	<0.001	0.142
≥70 (n = 74,802, 39.2%)	66,562	89.0	8240	11.0		
**Gender**						
Male (n = 104,219, 54.6%)	97,065	93.1	7154	6.9	<0.001	0.011
Female (n = 86,809, 45.4%)	81,307	93.7	5502	6.3		
**Cancer**						
Absent (n = 173,378, 90.8%)	163,173	94.1	10,205	5.9	<0.001	0.093
Present (n = 17,650, 9.2%)	15,199	86.1	2451	13.9		
**Unit**						
Surgery (n = 67,398, 35.3%)	66,146	98.1	1252	1.9	<0.001	0.207
Internal medicine (n = 95,364, 49.9%)	88,982	93.3	6382	6.7		
Intensive care (n = 28,266, 14.8%)	23,244	82.2	5022	17.8		

**Table 3:** Descriptive statistics and univariate analysis results according to the type of nutritional therapy.

	Enteral/Combined/Parenteral Nutritional Therapy (n = 8026, 63.4%)		Oral Supplement (n = 4630, 36.6%)			
	Frequency (n)	Percent (%)	Frequency (n)	Percent (%)	*p*-Value	Effect Size
**BMI**						
<20.50 (n = 2811, 22.2%)	1660	59.1%	1151	40.9%	<0.001	0.048
≥20.50 (n = 9845, 77.8%)	6366	64.7%	3479	35.3%		
**Weight loss within 3 months**						
Present (n = 6358, 50.2%)	3917	61.6%	2441	38.4%	<0.001	0.038
Absent (n = 6298, 49.8%)	4109	65.2%	2189	34.8%		
**Reduced dietary intake in the last week**						
Present (n = 7862, 62.1%)	5003	63.6%	2859	36.4%	0.513	0.006
Absent (n = 4794, 37.9%)	3023	63.1%	1771	36.9%		
**Severe illness**						
Present (n = 10,331, 81.6%)	7088	68.6%	3243	31.4%	<0.001	0.227
Absent (n = 2325, 18.4%)	938	40.3%	1387	59.7%		
**Impaired nutritional status**						
Absent (n = 3156, 24.9%)	1883	59.7%	1273	40.3%	<0.001	0.125
Mild (n = 4926, 38.9%)	3045	61.8%	1881	38.2%		
Moderate (n = 2573, 20.3%)	1553	60.4%	1020	39.6%		
Severe (n = 2001, 15.8%)	1545	77.2%	456	22.8%		
**Severity of disease**						
Absent (n = 1757, 13.9%)	752	42.8%	1005	57.2%	<0.001	0.305
Mild (n = 4810, 38%)	2593	53.9%	2217	46.1%		
Moderate (n = 2959, 23.4%)	2015	68.1%	944	31.9%		
Severe (n = 3130, 24.7%)	2666	85.2%	464	14.8%		
**Age**						
<70 (n = 4416, 34.9%)	2657	60.2%	1759	39.8%	<0.001	0.049
≥70 (n = 8240, 65.1%)	5369	65.2%	2871	34.8%		
**Gender**						
Male (n = 7154, 56.5%)	4444	62.1%	2710	37.9%	0.001	0.031
Female (n = 5502, 43.5%)	3582	65.1%	1920	34.9%		
**Cancer**						
Absent (n = 10,205, 80.6%)	6640	65.1%	3565	34.9%	<0.001	0.07
Present (n = 2451, 19.4%)	1386	56.5%	1065	43.5%		
**Unit**						
Surgery (n = 1252, 9.9%)	723	57.7%	529	42.3%	<0.001	0.253
Internal medicine (n = 6382, 50.4%)	3368	52.8%	3014	47.2%		
Intensive care (n = 5022, 39.7%)	3935	78.4%	1087	21.6%		

**Table 4:** Variables for models.

NRS-2002	Modified NRS-2002
BMI ≥ 20.5	BMI ≥ 20.5
Weight loss within 3 months	Weight loss within 3 months
Reduced dietary intake in the last week	Reduced dietary intake in the last week
Severe illness	Severe illness
Impaired nutritional status (absent, mild, moderate, severe)	Impaired nutritional status (absent, mild, moderate, severe)
Severity of disease (absent, mild, moderate, severe)	Severity of disease (absent, mild, moderate, severe)
Age ≥ 70	Age ≥ 70
	**Gender**
	**Cancer**
	**Unit (surgical unit, internal medicine unit, ICU)**

**Table 5:** Validation set performances of models for step 1.

Method	RF		ANN		DL		EN		NB	
Variable Set	NRS-2002	Mod NRS-2002	NRS-2002	Mod NRS-2002	NRS-2002	Mod NRS-2002	NRS-2002	Mod NRS-2002	NRS-2002	Mod NRS-2002
AUC (95% C.I.)	0.921 (0.916–0.926)	0.921 (0.916–0.926)	0.923 (0.918–0.929)	0.930 (0.925–0.935)	0.928 (0.923–0.933)	**0.933** **(0.928–0.937)**	0.929 (0.924–0.934)	0.932 (0.927–0.937)	0.927 (0.922–0.932)	0.928 (0.923–0.933))
Accuracy	0.939	0.937	0.925	0.931	0.935	0.926	0.920	0.926	0.930	0.932
Balanced Accuracy	0.579	0.547	0.797	0.801	0.782	**0.828**	0.826	0.826	0.792	0.777
MCC	0.308	0.241	0.504	0.527	0.522	**0.539**	0.520	0.536	0.513	0.505
Sensitivity	0.165	0.098	0.650	0.650	0.606	0.716	0.718	0.710	0.634	0.599
Specificity	0.994	0.997	0.944	0.951	0.959	0.941	0.934	0.941	0.951	0.955
PPV	0.652	0.671	0.452	0.487	0.510	0.463	0.435	0.461	0.477	0.489
NPV	0.944	0.940	0.974	0.975	0.972	0.979	0.979	0.979	0.973	0.971
F1 Score	0.264	0.172	0.534	0.557	0.554	**0.562**	0.542	0.560	0.544	0.538

**Table 6:** Validation set performances of models for step 2.

Method	RF		ANN		DL		EN		NB	
Variable Set	NRS-2002	Mod NRS-2002	NRS-2002	Mod NRS-2002	NRS-2002	Mod NRS-2002	NRS-2002	Mod NRS-2002	NRS-2002	Mod NRS-2002
AUC (95% C.I.)	0.689 (0.668–0.710)	0.703 (0.683–0.723)	0.704 (0.676–0.718)	0.710 (0.680–0.721)	0.716 (0.696–0.736)	**0.739** **(0.724–0.763)**	0.705 (0.685–0.726)	0.717 (0.70–0.737)	0.677 (0.656–0.698)	0.687 (0.667–0.708)
Accuracy	0.595	0.594	0.605	0.612	0.585	**0.665**	0.628	0.629	0.573	0.589
Balanced accuracy	0.638	0.651	0.646	0.645	0.645	0.687	0.654	0.659	0.631	0.646
MCC	0.275	0.311	0.289	0.283	0.301	**0.360**	0.299	0.309	0.271	0.301
Sensitivity	0.479	0.437	0.493	0.522	0.422	0.606	0.558	0.546	0.414	0.431
Specificity	0.798	0.865	0.799	0.768	0.868	0.768	0.751	0.772	0.848	0.862
PPV	0.804	0.849	0.810	0.796	0.847	0.819	0.795	0.806	0.825	0.844
NPV	0.469	0.470	0.476	0.481	0.464	0.529	0.495	0.495	0.455	0.466
F1 Score	0.600	0.577	0.613	0.631	0.563	**0.696**	0.655	0.651	0.552	0.571

**Table 7:** Absolute and relative differences between NRS-2002 and modified NRS-2002 models of DL.

	Step 1				Step 2			
Variable Set	NRS-2002	Modified NRS-2002	Increase/Decrease Absolute Effect (%)	Increase/Decrease Relative Effect (%)	NRS-2002	Modified NRS-2002	Increase/Decrease Absolute Effect (%)	Increase/Decrease Relative Effect (%)
AUC	0.928	0.933	0.006	0.60%	0.716	0.739	0.023	3.27%
Accuracy	0.935	0.926	−0.009	−0.98%	0.585	0.665	0.080	13.64%
Balanced Accuracy	0.782	0.828	0.046	5.89%	0.464	0.529	0.065	13.96%
MCC	0.522	0.539	0.017	3.29%	0.645	0.687	0.042	6.46%
Sensitivity	0.606	0.716	0.110	18.13%	0.301	0.360	0.060	19.91%
Specificity	0.959	0.941	−0.018	−1.84%	0.422	0.606	0.184	43.57%
PPV	0.510	0.463	−0.047	−9.27%	0.868	0.768	−0.100	−11.57%
NPV	0.972	0.979	0.007	0.76%	0.847	0.819	−0.028	−3.36%
F1 Score	0.554	0.562	0.008	1.50%	0.563	0.696	0.133	23.62%

**Table 8:** Test set performances of selected models.

	Step 1	Step 2
AUC (95%)	0.933 (0.926–0.936)	0.741 (0.722–0.761)
Accuracy	0.923	0.651
Balanced accuracy	0.828	0.681
MCC	0.530	0.352
Sensitivity	0.937	0.569
Specificity	0.719	0.794
PPV	0.979	0.827
NPV	0.449	0.515
F1 Score	0.958	0.674

## Data Availability

The data presented in this study are available on request from the corresponding author. The data are not publicly available due to privacy or ethical restrictions.
